# How “green” can religions be? Tensions about religious environmentalism

**DOI:** 10.1007/s41682-021-00070-4

**Published:** 2021-10-01

**Authors:** Jens Koehrsen, Julia Blanc, Fabian Huber

**Affiliations:** 1 Center for Religion, Economy and Politics, University of Basel, Nadelberg 10, 4051 Basel, Switzerland; 2 Theological Faculty, University of Oslo, Oslo, Norway; 3 Centre for Advanced Studies “Multiple Secularities”, University of Leipzig, Leipzig, Germany

**Keywords:** Environment, Ecology, Sustainability, Climate Change, Religion, Spirituality, Tensions

## Abstract

Scholarship has suggested a “greening” of religions, supposing that faith communities increasingly become environmentally friendly and use their potentials to address environmental challenges. This contribution points to the problems of the supposed “greening” by indicating the ongoing disagreements in many religious traditions over environmental engagement. The disagreements show that religious environmentalism is an embattled terrain that involves actors with different interests, backgrounds, and understandings of their traditions. The authors illustrate that tensions are an inherent part of religious environmentalism, becoming manifest in different views and theologies, ambivalences, misunderstandings, and sometimes mistrust. They distinguish between four types of tensions: (1) intradenominational tensions, (2) interdenominational tensions, (3) interreligious tensions, and (4) religious-societal tensions. By drawing attention to the tensions of religious environmentalism, this contribution sheds light on the struggles and limitations that religious environmentalists face in their ambitions to address climate change and other environmental challenges.

## Introduction

Against the backdrop of climate change and other environmental degradation processes, scholarship supposes that the world’s major faith traditions are becoming increasingly concerned about the natural environment. It has framed this religious innovation process as a “greening” of religions. This “greening” becomes visible in eco-theologies and spiritualities centering on the environment, environmental protection activities of local religious communities, and public statements of religious leaders, drawing attention to ecological problems and conveying support for pro-environmental policies. A prominent example of this is Pope Francis’ *Laudato Si’ *(Pope Francis 2015). And also many other leaders and faith communities have launched public statements such as the *Islamic Declaration on Global Climate Change* (International Islamic Climate Change Symposium 2015) or the publications of the “green” Patriarch Bartholomew of Constantinople (Bartholomew and Chryssavgis 2012; Theokritoff 2017).

Given its strong public visibility, in particular, *Laudato Si’ *has generated strong expectations about a major sea change in society and in the Catholic Church. Climate scientists, media representatives, and Catholic environmentalists applauded the encyclical, and some supposed a “Francis effect,” claiming that the encyclical would lead to a massive rise in public concern about climate change (Maibach et al. 2015). As the very leader of this hierarchical religious organization urged humanity to address the increasing environmental challenges, it seemed to constitute a powerful sign of a “greening” of the Catholic Church. Consequently, Catholic environmentalists interpreted *Laudato Si’* as the strongest possible support of their cause and expected their position in the Church to improve substantially. However, the supposed sea change did not occur. Catholic environmental groups received neither more resources nor a stronger voice in their national churches. Also the impact of *Laudato Si’* on the climate activism of the Church and its adherents has remained unclear (Woodworth 2020). Rather than creating a joint environmental program, the encyclical rendered visible disagreements about the environmental orientation in the institution. Many segments of the Church (e.g., Bishops, local churches, members) disagreed with the contents of Pope Francis’ environmental program, questioned the relevance of* Laudato Si’*, and only supported it to varying degrees (Li et al. 2016; Landrum et al. 2017; Vincentnathan et al. 2016).

This example illustrates the tensions of religious environmentalism. It stands for the ongoing disagreements in many religious traditions over environmental engagement (Koehrsen and Huber 2021). While many scholars have highlighted the potentials of religions to address environmental challenges and suggested a “greening” of religions (Chaplin 2016; Tucker 2006; Tucker and Grim 2016; Palmer 2013), the tensions show that religious environmentalism is an embattled terrain that involves actors with diverging interests, backgrounds, and understandings of their traditions. There is neither a linear direction of “greening” nor a univocal impact on society.

Tensions appear when an actor or institution represents views or values that contrast those of other actors or institutions. They can become manifest in disagreements or frictions. In the field of religion, tensions may, for instance, become apparent as divergent interpretations of the given tradition or as power struggles in a religious organization with different camps seeking to determine the future direction of the given organization (Zuckerman 1997; Roof and McKinney 1987). In other occasions, tensions may emerge between organizations that compete for member appeal, resources, and social status (Miller 2002; Pyle and Davidson 2014; Chesnut 2003) or as an antagonism between the values of a religious community and its social environment (Iannaccone 1994).

This contribution suggests that tensions are an inherent part of religious environmentalism. These usually do not manifest themselves in open clashes between different parties but in different views and theologies, ambivalences, misunderstandings, and sometimes mistrust. Such tensions can create effective barriers for religious environmentalism. Thereby, this contribution sheds light on the problems of the supposed “greening” of religions as well as on the limitations that religions face when they seek to address environmental degradation. In this article, we will provide an analytical scheme that situates these tensions at different levels, distinguishing between (1) intradenominational tensions, (2) interdenominational tensions, (3) interreligious tensions, and (4) religious-societal tensions.

The remainder of this article is structured as follows. The first part introduces the reader to the notion of the “greening” of religions and their strong potentials to address environmental challenges. The following section contrasts this optimistic picture by accentuating empirical insights that point to the challenges of religious environmentalism. These show that empirical evidence does not support the assumed “greening” of religions. Bringing both perspectives together, the next section discusses the tensions of religious environmentalism. Here, we portray the aforementioned types of tensions and illustrate each of them with empirical examples. Finally, the conclusion relates our focus on tensions to broader debates on religious innovations and the role of religions in societal challenges, and suggests avenues for future research.

## Green religious optimism

The academic debate on religion and ecology has been the focal point for endeavors to study religious environmentalism (Berry 2013). Lynn White’s (1967) seminal article in the journal *Science* has been an important starting point for this debate (for a review of earlier academic contributions asserting a negative impact of Abrahamic traditions on nature, see Taylor 2016, pp. 277–286). In this article, White argued that Western Christianity with its anthropocentrism caused the environmental crisis. Interestingly, at the end of the article, he claimed that religions need to be part of the solution for the crisis: “More science and more technology are not going to get us out of the present ecologic crisis until we find a new religion, or rethink our old one.” (White 1967, p. 1206)

White’s contribution was an important starting point for academic debates about “ecology and religion” as well as for “greening” efforts within Christianity (Whitney 2017). Partly in response to his criticism, Christian theologians as well theologians from other faith traditions have sought to provide ecological reinterpretations of their traditions and to generate faith-based environmental ethics that address the ecological crisis (Tucker 2006; Blanc 2017; Foltz 2006; Dessi 2013; Harris 1995; Binay and Khorchide 2019; Boff 2011; Ostheimer and Blanc 2021). Some scholars (e.g., Chaplin 2016; Tucker 2006, 2008) have interpreted these endeavors as leading to a “greening” of religions, thus suggesting that religions become more environmentally friendly over time.

At the same time, contributions from the religion and ecology debate have outlined the potentials of religions to address environmental problems (Gardner 2003; Gottlieb 2010; Peterson 2007). Given that more than 80% of the global population are part of a religious tradition (Pew Forum 2015), religions can reach broad population segments. Through their worldviews and ethical teaching, they can shape the lifestyles of their adherents and their relationships to the natural environment (Mangunjaya and McKay 2012; Sheikh 2006; Jenkins 2009; Watson and Kochore 2012). As such, Tucker writes: “religions can encourage values and ethics of reverence, respect, redistribution, and responsibility for formulating a broader environmental ethics that includes humans, ecosystems, and other species. With the help of religions humans are now advocating for a reverence for the earth (…).” (Tucker 2006, p. 401) Moreover, religious leaders and umbrella organizations often enjoy a high public credibility and sometimes have close relationships with societal decision-makers (e.g., politicians, business elites) (Casanova 1994; Davie 2010). They can draw on their networks and prominence to influence public debates, create awareness for environmental problems, and influence decision-making processes (Reder 2012; Schaefer 2016; Wardekker et al. 2009). Furthermore, many religious organizations have vast financial resources and infrastructures (e.g., buildings, teaching facilities) at their disposal that they can employ to support societal transformations towards environmental sustainability (Gardner 2002, 2003; Palmer 2013; Blanc and Ostheimer 2019). These views represent, of course, an optimistic narrative of the potentials of religious communities. We will address their limitations in the coming sections.

Apart from the major global faith traditions, scholarship has pointed to indigenous religions and new spiritualities that go hand in hand with environmental protection (for a comprehensive overview, see Taylor et al. 2016a, pp. 335–347). Though these tend to be less visible than the dominant faith traditions (Luckmann 1967), they may be equally or even more effective in promoting care for the environment (Taylor 2004; Naess 1990; Hedlund-de Witt 2012, 2013; Sponsel 2012). For instance, Bron Taylor (2010) has suggested the emergence of a new world religion that he calls “Dark Green Religion.” It considers the natural environment as sacred, involves feelings of connectedness to nature, and promotes an ethics of reverence for nature. “Dark green religion considers nonhuman species to have worth, regardless of their usefulness to human beings. Such religion expresses and promotes an ethics of kinship between human beings and other life forms.” (Taylor 2008, p. 89) Taylor argues that this new eco-spirituality is already an essential part of the environmental milieu. Therefore, one may assume that civil-society groups that engage in environmental protection (e.g., Extinction Rebellion) but also holistic life-style movements (e.g., Anthroposophy) have incorporated this eco-spirituality. Aside from Taylor, other scholars (e.g., Hedlund-de Witt 2013; Johnston 2014; Nelson 2012; Witt 2016) have highlighted the underlying religious dimensions of environmentalism and of concepts related to it (e.g., sustainability).

Additionally, literature on religion and ecology frequently depicts indigenous cosmologies as pro-environmental. Many of these cosmologies conceptualize non-human beings (such as animals and plants) as part of the social universe, demand respectful behavior towards them, and prefigure potential sanctions in case of non-conformance (Lockhart et al. 2019; Castro 1998; Howell 2012). For instance, scholars have stressed that some values from African traditional religion encourage reverence for the natural environment (Eneji et al. 2012; Ikeke 2015; Jimoh et al. 2012; Kelbessa 2005; Le Grange 2012; Warner and Hoskins 2008). Communities related to these values conceive sacred forests as habitats of deities and, therefore, consider deforestation as a taboo (Teka et al. 2013; Chisadza et al. 2015). While many contributions provide empirical examples of the pro-environmental tendencies of communities related to indigenous traditions (Cox et al. 2014; Tiedje 2008; Martinez 2018), others disagree and find little empirical support for this (Lorentzen and Leavitt-Alcantara 2006; Descola 2005). An important variable is the degree to which these communities remain culturally and economically intact. As indigenous communities increasingly exchange with their social environment and are subject to the pressures of neo-liberal capitalism, traditional lifestyles and knowledge systems change, leading these communities to diverge from their original nature-based lifestyles (Gabbert 2018; Kent 2010).

While the relationship between ecology and religion has become an increasingly prominent topic in religious studies, environmental studies and climate change research have more recently started to regard religions as an asset for addressing environmental challenges (e.g., Haluza-DeLay 2014; Jenkins et al. 2018; Edenhofer et al. 2015; Allison 2015; Smith and Leiserowitz 2013; Kilburn 2014; Koehrsen 2018c, 2021). Representatives from both academic fields—environmental studies/climate change research as well as the religion and ecology debate—have pointed to the potentials of religion, arguing that religions could make a significant difference in addressing environmental challenges such as climate change.

At the same time, we can witness an increase of environmental activism in different faith traditions. This becomes prominently illustrated by the abovementioned encyclical *Laudato Si’*, the* Islamic Declaration on Global Climate Change* or the *Interfaith Climate Change Statement to World Leaders*, and many other public statements issued by religious leaders, umbrella organizations, and international religious networks. These statements appear as evidence for the “greening” of religions. They indicate a rising interest in environmental issues among religious leaders, umbrella organizations, and networks. However, it remains unclear what actions follow from such statements and whether local faith communities and their adherents become “greener” to the same extent.

## Challenges for the religious greening

Contrasting the optimistic pictures painted by sections of the religion and ecology debate, social science research has pointed to different challenges of religious environmentalism. These relate (a) to the absence of the supposed “greening” of religions and (b) to religious practices and worldviews that cause environmental harm.

Multiple studies have explored the assumed “greening” of religions (for comprehensive reviews of existing empirical research, see Taylor 2016; Taylor et al. 2016a). Quantitative analyses about environmental attitudes among adherents of specific faith communities show no clear evidence for a “greening” (Carlisle and Clark 2018; Clements et al. 2014; Konisky 2018; Dilmaghani 2018). Accordingly, in a review of existing quantitative research on the “greening” of Christianity in the US, Bron Taylor et al. (2016b, a) conclude that empirical research does not support the “greening” hypothesis: the environmental attitudes of religious followers do not reflect a potential “greening” of their religions. Despite the emergence of green theologies, climate change and other environmental problems constitute challenges to religious organizations to which they respond in different ways (Jenkins et al. 2018). Consequently, there is no encompassing and straight development path towards a “greening” in religions (Haluza-DeLay 2014).

Apart from the aforementioned problems in the “greening,” studies have also found that religious practices can involve environmental harm, as specific rituals produce negative ecological impacts (Wexler 2016). Prominent examples are the carbon emissions and waste created by religious pilgrims worldwide.

Moreover, religious views can discourage adherents from actively addressing environmental problems. These views can involve (a) skepticism about the existence of environmental problems, (b) the perception of the problems as a welcomed end-of-times, or (c) as divine punishments for human sins. In terms of skepticism about environmental problems, a prominent topic is climate change denial, often reported to be prevalent among sections of Christianity (Zaleha and Szasz 2015; Veldman 2019; Carr et al. 2012; Hayhoe et al. 2019). However, among Muslims, we can also find groups that regard “climate change” as “poisonous knowledge from the West” (Khan 2014) or as a Western conspiracy to weaken Muslim-majority countries (Yildirim 2016). Apart from skepticism about climate change, its acceptance as a sign of the looming end-of-times constitutes a hindrance for climate engagement (Nche 2020; Barker and Bearce 2013; Artur and Hilhorst 2012). In this view, humans are not responsible for climate change given that it is the fulfillment of an end-time prophecy. From this perspective, believers are likely to welcome and embrace climate change. Finally, a third interpretation that does not encourage direct environmental action perceives ecological problems as a divine punishment for human sins. In this perspective, God responds with different forms of environmental degradation or disasters to the sinful behavior of political leaders (e.g., corruption) or local populations (e.g., stealing, lying). Research has reported this view, for instance, for different regions of Sub-Saharan Africa (Bell 2014; Abegunde 2017; Haron 2017). Apart from (evangelical) Christianity and Islam, these views may become manifest in other religious traditions.

These challenges relate to the tensions that we want to stress and contrast the sometimes romantic views on religious earth stewardship.

## Tensions in religious environmentalism

Research on religious environmentalism has tended to explore how religion matches or mismatches environmental protection. In so doing, it often suggests a forthright relationship between religion and the environment, leading to environmental protection (Gardner 2003; Tucker 2006, 2008), a neglect of the environment (Taylor et al. 2016b), or even environmental degradation (White 1967; Barker and Bearce 2013; Amery 1974). However, to understand the multifaceted ways in which religions position themselves vis-a-vis the environment and the ambivalences underlying their positions, there is a need to study the tensions inherent in religious environmentalism.

Environmentalism is an embattled terrain. This becomes, for instance, visible in the variety of narratives around environmental sustainability. Given that actors promote diverging understandings of sustainability, there is no generally accepted definition (Berger et al. 2014; Bergman et al. 2015a, b; Luederitz et al. 2016; Garud and Gehman 2012; Lockie 2016; van den Bergh et al. 2011; Neckel 2017, 2018). Originating from forestry and having internationally been coined by the Brundtland Report in 1987 and Earth Summit in 1992, sustainability has become an increasingly popular term (Grober 2007; Lele 1991; Mebratu 1998; World Commission on Environment and Development 1987). Transformation projects on different socio-geographical scales and by different actors are increasingly labeled as “sustainable.” Based on their background and interests, actors hold different views about the shape of these transformation processes. From a sociological perspective, this plurality allows for the study of different notions of sustainability in specific contexts as well as among particular types of actors. Against this background, we abstain from imposing a definition that could potentially disqualify the notions and activities of some of the actors studied as not belonging to “sustainability.”

Regardless of the concrete understanding of “sustainability,” sustainability transitions endeavor to contest existing structures and routines to varying degrees. They seek to transform established structures and routines towards configurations that are regarded by the given actors as more “sustainable.” Due to this and the fact that actors subscribe to competing transition narratives, such transformations are marked by power struggles over their shape and are, therefore, deeply political (Gabillet 2015; Rutherford 2014; Meadowcroft 2009; Koehrsen 2018b). These struggles do not only concern disagreements between environmentalists and actors interested in maintaining the status quo (e.g., carbon-based fuel industry) but also between promoters of environmental sustainability that hold different views on the appropriate transformation approach (e.g., technological vs. cultural approaches, see O’Brien 2018; Luederitz et al. 2016).

These tensions also become apparent in the domain of religious environmentalism, as it implies innovation processes within religions as well as in the broader societies in which religions are embedded. As described above, the innovation processes entail different visions of the changes and, therefore, lead to tensions. By undertaking empirical research on tensions, we can grasp these oppositions and the negotiation processes that take place around the supposed “greening” of religions. In this way, we gain insights into the ongoing “(non)greening” processes in different types of religious communities and at different organizational levels (e.g., national umbrella organizations and local congregations). Studying such tensions, scholarship can generate a more nuanced picture of religious environmentalism. Religious environmentalism is neither a linear nor a smooth process that finally leads to the “greening” of a given tradition and its social environment. Paralleling other types of environmental engagement, religious environmentalism faces resistance, limitations, and conflicts while seeking to change the existing order. Sometimes, said tensions can be located in the fabric of religious organizations, in the form of counter movements, rivaling theological schools, or simply a lack of financial resources, obstructing internal transformations and tearing to pieces the prospects of a “greening” process. In other cases, rivalry between different religious traditions, resistance of non-religious actors, the obduracy of secular institutions, or the marginalization of religion can thwart the impact of religious environmentalism. The tensions of religious environmentalism can become manifest on four different levels: (1) intradenominational tensions, (2) interdenominational tensions, (3) interreligious tensions, and (4) religious-societal tensions. In the following, we illustrate each of these types by drawing on examples from studies in different world regions. These studies are taken from the literature on religious environmentalism and have been conducted by researchers from different disciplinary backgrounds (e.g., anthropology, political sciences, religious studies, sociology, theology). Many of them are part of a volume on the tensions in religious environmentalism (Koehrsen et al. 2021).

### Intradenominational tensions: struggles and institutional barriers within communities

Intradenominational tensions occur within a specific religious denomination or branch/group. These can become, for instance, visible as frictions between ethical teachings and deeds, between different understandings of the teachings and the environmental problems within the same religious community, or between the environmental ambitions of some members and the hierarchal structures of the given religious organization. Often, such oppositions are overseen in presenting, for example, “the” Christian or “the” Muslim view on ecology. Small-scale findings on internal oppositions are precious for understanding the broader dynamics of religious environmentalism. Not only do they show that frictions emerge even within small groups, but also that they are a fundamental part of religious environmentalism.

Research shows that even when religious head offices decide on “greening” strategies and undertake programs to improve the environmental sustainability of the given organization, congregations and branches do not necessarily implement these activities (Amri 2014; Vaidyanathan et al. 2018; Torabi and Noori 2019; Koehrsen 2015). “Greening” programs face resistance in religious organizations. Research on mainline Christian churches in the German-speaking parts of Switzerland illustrates this resistance (Koehrsen and Huber 2021; Huber and Koehrsen 2020): umbrella organizations seek to push environmental sustainability, while local congregations often oppose these green policies of their head organizations. The head organizations have designed environmental programs, new regulations and ecological support schemes for their local congregations. However, only a few Catholic and Protestant congregations actively participate in environmental protection. Even when obliged by their umbrella organizations to adapt to more environmentally friendly standards, congregations may still find ways of avoiding to implement these. For instance, a religious head organization supplied their local congregations with new, more environmentally friendly cleaning products. However, many congregations just moved these new cleaning products away and continued to work with the old ones. Learning about this, officers of the head organization withdrew the old cleaning products from the congregations and requested them to work only with the new products. Nevertheless, some congregations still circumvented this request and continued to use the old products that they had secured in hidden storages from the officers. Umbrella organizations sometimes face strong resistance in implementing more environmentally friendly measures in their congregations. The aforementioned research (Koehrsen and Huber 2021; Huber and Koehrsen 2020) relates these frictions to different priorities at the level of congregations and umbrella organizations. While umbrella organizations address the public image of the given denomination and, therefore, increasingly engage in environmental sustainability, congregations tend to avoid distractions from their focus on their individual members and congregational core activities.

Similarly, a study by Christophe Monnot (2021) shows frictions within Swiss churches. However, in this case, the environmental engagement from below faces institutional barriers at the higher levels of the religious organization. The study describes cases of two congregations strongly committed to environmental activism: a Catholic parish and a reformed congregation. The cases illustrate that the congregations face organizational challenges within the hierarchies of their denominations in their endeavor to “green” the given cantonal churches, as the organizational structures do not allow for broad changes in the churches. Such frictions in denominational bodies have also been reported by other studies (Vincentnathan et al. 2016; Shibley and Wiggins 1997; Zaleha and Szasz 2015).

Sometimes these frictions are related to different camps within religious or spiritual organizations. These camps promote competing understandings of environmental sustainability. As such, a study reveals different positions regarding environmental commitment within the same Roman Catholic orders (Gojowczyk 2020, 2021). While some prioritize a scientific approach, others emphasize social work or put forward a spiritual approach. Moreover, they disagree on whether they should direct environmental actions inwards or outwards. The positions the different actors take depend on the specific roles that they assume in the orders and their professional background. Another study shows tensions within a Swiss agricultural movement related to anthroposophy (Majerus 2021). Older, strongly anthroposophical farmers hold anthropocentric views, placing human beings at the center of their agricultural activities. By contrast, novices question this anthropocentrism and orient themselves towards other eco-spiritual strands that share elements of deep ecology or dark green religion. The differing views between the established anthroposophical farmers and the newcomers led to struggles over how agriculture should be oriented.

These examples show that institutional barriers, competing views, and power struggles within religious and spiritual groups create tensions within religious environmentalism.

### Interdenominational tensions: dissonance between communities from the same faith-Background

Interdenominational tensions are those that unfold between different denominations or branches of the same religion. Different branches and communities that belong to the same religious tradition may pursue different ecological agendas and disagree on their understandings of environmental challenges such as climate change, and thus may promote divergent responses to it. A prominent example is the smoldering conflict between different camps of the evangelical movement in the US with regard to the government’s climate policy (Carr et al. 2012; Wardekker et al. 2009; Nagle 2008; Veldman 2019; McCammack 2007).

These tensions also emerge in other traditions, such as Islam (Koehrsen 2021). A study of halal wastewater recycling in Indonesia sheds light on the different positions that the three biggest Muslim organizations in this country assume with regard to the re-use of wastewater (Jamil 2021). Thereby, these organizations address the question whether and under what conditions water recycling is halal. Whereas the *Majlis Ulama Indonesia* (a state-supported umbrella organization of Islamic scholars and clerics) and *Muhammadiyah *(the representatives of a “modernist” position) position themselves in favor of re-use, *Nahdahtul Ulama* (the “traditionalist” camp with strong influence in rural areas) opposes the recycling of wastewater and its re-use for ritual ablution or consumption. The study illustrates that there is not “one Islamic” view on water recycling. Different currents within Islam will favor distinct visions on environmental issues.

Also in Nigeria, we can find divergent environmental views within one single religious tradition. For instance, research by George Nche indicates disagreement among Christian leaders (Nche 2021, 2020). Some of them question the anthropogenic dimensions of climate change and argue that Christians should focus on soul winning and Parousia. Another form of interdenominational tension are disparities between theology and practice. A study indicates major gaps between eco-theology in the Christian mainline discourse and the action in local congregations (Blanc 2021). The low environmental commitment of many churches contrasts the high eco-theological ideals and illustrates the difficulties of disseminating eco-theological concepts within religious communities. While the results are equally low in Catholic and Protestant/Reformed congregations (with some explicit exceptions), the normative-theoretical background between Roman-Catholics and Protestants differs.

### Interreligious tensions: competition and boundary drawing between different faith traditions

Religious environmentalism has been regarded as a suitable topic for interreligious dialogue and, therefore, for bringing actors from different traditions together to establish joint grounds for enduring interfaith partnerships. Nevertheless, representatives of different religious traditions may hold different views on environmental problems and the role of religions in addressing them, thereby potentially creating interreligious tensions. This can also bring prejudices against each other to the surface and, therefore, involve tensions. An investigation on the interreligious cooperation “Religious Week of Nature Conservation” in Germany illustrates these tensions (Dohe 2021, 2020). Religious organizations from different faith backgrounds (i.e., Baha’i, Buddhist, Catholic, Muslim, Protestant, Sikh) collaborated to organize various events around the topic of nature conservation during one week. However, religious prejudices became apparent in this interreligious consultation process, as many non-Muslim participants were suspicious vis-à-vis the Muslim partners, questioning the credibility of their environmental engagement. These struggled to overcome these “reservations” in the light of nature protection.

Sociological field models can help to explain such interreligious tensions by stressing the competition between religious groups (Koehrsen and Huber 2021; Huber 2021). When environmental sustainability assumes an increasing importance in public agendas, credible engagement in this topic may become an asset in struggles for societal legitimacy. As such, established religious actors and newcomers may compete over the credibility of their environmental activities. They aim to become pioneering “green” religions in their given societies, as this can lead to an advantage in their public perception and social legitimacy. Tensions between the religious actors may become manifest in the competition. These do not arise from environmental commitment itself but relate to the competition between religious organizations over societal legitimacy, religious authenticity, and access to resources. Taylor et al. indicate a similar direction in their review of existing research on religious environmentalism when stating: “It may be, therefore, that some religious environmentalism reflects a strategy to retain or gain members by getting in synch with trending values and concerns.” (Taylor et al. 2016a, p. 350).

In other cases, religious communities may negotiate their relationship to competing faith traditions. As such, a study reveals how an African-initiated church in South Africa, the Zion Christian Church, relates to African traditional religions in its endeavor to address environmental problems by developing a new theology (Stork 2021). On the one hand, it draws on concepts from African traditional religions; on the other hand, it aims to distance itself from these. In this way, environmental commitment ultimately reveals itself as a negotiation process that involves balancing between different concepts and the drawing of symbolic boundaries to demarcate the unique identity of the own community.

### Religious-societal tensions: oppositions and negotiations between religion and society

Religious-societal tensions refer to frictions between religion(s) and their given societal environment. Religious groups and societal actors (e.g., secular environmental groups) may struggle with each other over the shaping of climate policies. At the same time, societies may create barriers that inhibit the engagement of religious actors in environmental affairs (e.g., exclusion of religious actors or religious reasoning from specific institutions), while secular environmental groups may stigmatize specific religious groups or religion in general.

Research on religious actors’ participation in the United Nations climate negotiations illustrates such tensions. Studying faith-based organizations at the United Nations (UN), Jeffrey Haynes has described the UN as a “demonstrably secular organization, founded on nonreligious values” (Haynes 2014, p. 24) and based on “secular-liberal ethos and foundations” (Haynes 2014, pp. 6–7). Engaging in such a secular sphere generates tensions for religious actors (Glaab 2017, 2021; Krantz 2021). These employ two different strategies to deal with these tensions: (a) adaptation to the secular field by concealing the religious identity or (b) demarcation of the religious background. However, the choice of the strategy ultimately also depends on the concrete context: as such, religious actors tend to downplay the religious background in meetings with secular actors and emphasize their religiosity in interfaith meetings. Similarly, other secularized arenas of environmental engagement lead to a hiding of religious identities. For instance, in Germany’s energy transition processes, environmental actors avoid revealing their spiritual motivations (Koehrsen 2018a). Fearing stigmatization, they perceive that there is no place for spirituality in these transformation processes which are strongly shaped by technological rationality.

In some cases, religious actors also engage in favor of environmentally unsustainable solutions and, therefore, face backlashes from the broader society. An example of this is kosher electricity (Herman 2021). A rising number of Israeli ultra-orthodox communities employ different electricity-sources on Shabbat and other Jewish holidays to avoid the work of Jews during these days since it is regarded as unkosher. To produce kosher electricity, the communities tend to use diesel generators during these days. However, this use causes several tensions. Environmentalists criticize unsustainable kosher electricity solutions, and the state has prohibited the use of diesel generators given their negative side effects (e.g., pollution, accidents). Yet, most ultra-orthodox communities are against renewable energies because they are expensive and difficult to implement.

Tensions with the broader society emerge when religious groups show no environmental engagement or even engage in polluting practices and, therefore, do not fulfill the societal expectations in terms of their earth-caring engagement (Harmannij 2021).

Nevertheless, some religious actors also seek to distance themselves from the broader society, which they perceive as damaging the planet, and, therefore, draw symbolic—and sometimes even geographic—boundaries between themselves and the rest of society. An example of this are eco-spiritual communities such as biodynamic wine-crafters where some actors engaging with Rudolf Steiner’s cosmological legacy seek distinction from “normal” sustainable agriculture (Grandjean 2021).

## Conclusion

Tensions in religious environmentalism shed a critical light on the optimistic perspectives in the religion and ecology debate that stress global “greening” processes. The tensions illustrate that there is no linear religious “greening” process among the world’s faith traditions. Instead, we can find “greening” tendencies that struggle against “ungreen” oppositions as well as among each other. Studying the tensions of religious environmentalism helps to explain why the supposed “greening” faces barriers and diffuses only slowly within religious traditions or is not taking effect at all. These can be institutional barriers in the given organization or related to the power of traditionalist camps. However, even when members of a religious tradition agree on the importance of addressing environmental issues, tensions may become manifest in different ecological interpretations of the given tradition and lead to diverging practical approaches. Tensions may also evolve between different religious communities as well as between religions and their broader societal environment. Religious communities might compete over societal recognition, seeking to be regarded as authentically caring for the environment. At the same time, the societal environment could create barriers for the successful engagement of religious communities in public debates. For instance, it may establish mostly secular negotiation arenas that tend to exclude religious reasoning. But not only “secular” society might seek to ban religion from its public arenas. Religious actors, too, may distance themselves from mainstream society. Yet, even when aligning with mainline society, they might find it difficult to fulfill societal expectations with regard to their environmental engagement.

Despite these divergences and contentions, visible conflicts over religious environmentalism barely emerge. Tensions become manifest in different positions, ambivalences, misunderstandings, and sometimes mistrust. They lead to institutional barriers and challenges in “greening” endeavors, but they hardly erupt into visible clashes. However, by staying below the surface, tensions may become even more effective barriers for transformations. By remaining invisible, they can hardly be addressed in a direct manner and create frustration rather than enthusiasm for change.

The article has suggested four types of tensions. The individual types of tensions do not necessarily emerge in an isolated way. They are often interrelated, and boundaries between individual types of tensions may blur. For instance, when religious organizations are unable to implement pro-environmental policies due to internal frictions, they cannot fulfill broader societal expectations in terms of their environmental engagement (Harmannij 2021). In this case, internal religious tensions result in religious-societal tensions. From this perspective, addressing internal tensions appears fundamental not only to achieve internal cohesion but also to circumvent any peril to the social reputation and public image of the given religious organization. At the same time, religious-societal tensions may assume an important function for the “greening” processes, as societal pressure can facilitate innovation processes within religious traditions. Therefore, rising public concern over environmental degradation—as illustrated, for instance, by the Climate Strikes—seems to encourage the long-assumed “greening” processes of religious communities (Koehrsen and Huber 2021).

Additionally, the tensions of religious environmentalism are frequently connected to broader societal tensions around environmentalism. Therefore, the disagreements within the religious sphere appear to largely reflect those in other societal spheres where different camps support diverging approaches to address environmental challenges, with some promoting technological transitions, others political change, and again others deep cultural transformations (see also O’Brien 2018; Luederitz et al. 2016). Perhaps one of the most apparent differences to other social spheres is the supposed traditionalism of religion that portrays religions as hostile to change. Contrasting the supposed traditionalism, many of the studies quoted in this article point to innovation processes within religious traditions. Green religious “entrepreneurs” create transformation impulses. However, paralleling innovation processes in other social spheres, these green innovations need to negotiate their legitimacy in the context of predominant institutions (e.g., dogmas, rules, and hierarchies).

Scholars of religion need to address these tensions to understand the challenges of religious environmentalism. Future research may generate broader quantitative insights that measure the diffusion of specific “green” and “ungreen” theologies in different traditions and communities. Moreover, there is a need to strengthen research endeavors about the role of religions in environmental sustainability in the Global South. Given the importance of religion for the lifestyles and worldviews of broad population segments in many Global South societies, it is here where religion could make a fundamental difference. To get a better understanding of effects of religions on the environment in these regions, the academic study of religion should engage in collaborations with environmental studies and climate change research. Finally, the abovementioned interconnectedness of different types of tensions could constitute an insightful direction of research. Scholars might explore how different types of tensions of religious environmentalism relate to each other and to broader societal tensions around environmentalism.

Beyond the mere focus on the “greening” of religions, exploring tensions in religious environmentalism relates to two general topics in the academic study of religion: (a) innovation processes in religions and (b) the role of religions in societal transformation processes.

Tensions in religious environmentalism point to the challenges of religious innovation processes (Koehrsen 2019; Nagel 2018; Finke 2004). Ambitions to transform religions go hand in hand with the emergence of different visions with regard to the intended change. The diverging visions imply tensions that will affect the innovation process. The insights on religious environmentalism might also be applicable to other religious innovation processes—such as the evolution of European Islam, the rising digitalization of religions, or the diffusion of prosperity gospel within Pentecostalism—by pointing to the tensions inherent to these developments. Religious innovations face barriers, become negotiated and modified, and finally divert from the originally intended pathway.

Furthermore, tensions in religious environmentalism indicate how the relationship between society and religion is negotiated in the face of societal challenges, becoming visible in debates around “post-secularity” (Habermas 2008; Baker and Beaumont 2011; Casanova 2006; Berger 1999). This opens up the question of what position religious groups should assume in their societies: Should they address societal problems and contribute to societal transformation endeavors? Should they have a voice in debates around sustainability? These questions need to be addressed within the very religious communities as well as by their surrounding societies. On both sides, we can find different opinions as well as negotiation processes. Internally, religious communities sometimes struggle over the question whether they should participate in public debates and voice their views on political decision-processes. Externally, religious communities might face resistance as well. Global North societies sometimes generate considerable hindrances for the participation of religions in negotiations about the future development of their societies. By contrast, in other contexts, religious groups might become key stakeholders for societal transformations that could potentially block or promote the intended transition process. Religious and societal actors can contest existing secular-religious boundaries, aiming to shift, dissolve, or strengthen them. These processes can become visible when religious actors seek to engage in societal debates on sustainability or when societal actors strive for exploiting the potentials of religions for sustainability transitions. In both cases, existing secular-religious boundaries are likely to structure the engagement of religions in these societal transformation efforts. However, their involvement might also lead to negotiations about the role of religions in their societies that could affect these boundaries.
